# Rare combination of simple virilizing form of 21-hydroxylase deficiency, Graves’ disease and 47, XXX in a woman: A case report

**DOI:** 10.1097/MD.0000000000031443

**Published:** 2022-10-28

**Authors:** Dong Liang, Minmin Han, Linxin Xu, Yi Ren, Yi Zhang, Jianhong Yin, Jing Yang, Yunfeng Liu

**Affiliations:** a Department of Endocrinology, First Hospital of Shanxi Medical University, Shanxi Medical University, Taiyuan, China; b Department of Pharmacology, Shanxi Medical University, Taiyuan, China.

**Keywords:** 47, case report, congenital adrenal hyperplasia, simple virilizing form of 21-hydroxylase deficiency, Graves’ disease, XXX

## Abstract

Coexistence of congenital adrenal hyperplasia due to 21-hydroxylase deficiency, Graves’ disease and 47, XXX is rare. We report a case of a 25-year-old woman presented with masculine appearance, hirsutism and enlarged clitoris. Lab tests showed elevated serum 17 hydroxyprogesterone, testosterone, dehydroepiandrosterone. Gene test revealed heterozygous gene mutation in CYP21A2:NM_000500:exon4:c.518 T > A, NM_000500:exon8:c.C1024T. Karyotype analysis showed 47, XXX. After prednisone replacement and antithyroid therapy, she got a normal menstruation and normal level of testosterone. These findings demonstrate that patients with abnormal chromosome are likely to combine 21-hydroxylase deficiency (21-OHD), thus karyotyping test should not be neglected for those who have been already diagnosed as 21-OHD. Additionally, chromosomal abnormality such as 47, XXX and Turner syndrome had susceptibility to develop autoimmune thyroid disease because a gene on X chromosome may be responsible for the occurrence of autoimmune thyroid disease. Moreover, both 21-OHD and Graves’ disease (GD) can lead to high level of testosterone, thus we should keep in mind to test chromosome and thyroid function in 21-OHD patients to avoid misdiagnose or missed diagnosis. To the best of our knowledge, this is the first report of simple virilizing (SV) 21-OHD patient combined with 47, XXX and Graves disease.

**Patient concerns:**

A 24-years-old female of Han ethnicity was admitted to the endocrinology department complaining of absence of menses for half a year. The patient didn’t noticed her enlarged clitoris until she was 17 years old. Her menarche was 16 years old and the final height was 163 centimeter. She was diagnosed with GD 2 months before admission to our hospital due to palpitation, heat intolerance, muscle weakness.

**Diagnoses:**

The patient was diagnosed with SV 21-OHD, Graves disease and 47, XXX.

**Interventions:**

At first, the patient was given 10 mg methimazole twice a day as well as 5 mg predisone in the morning and 2.5 mg in the evening. After a year of regular medication and reexamination, she got a regular menstruation and thyroid function and now is taking 2.5 mg prednisone twice a day.

**Outcomes:**

The patient got a regular menstruation and thyroid function. Laboratory results showed: testosterone declined to 0.1nmol/L (0.1–1.67nmol/L) and 17 hydroxyprogesterone get back to normal level: 1.01ng/ml (0.30–2.34ng/mL). However, her enlarged clitoris has not narrowed.

**Lessons:**

Patients with abnormal chromosome are likely to combine 21-OHD, thus karyotyping test should not be neglected for those who have been already diagnosed as 21-OHD. Additionally, chromosomal abnormality such as 47, XXX and Turner syndrome had susceptibility to develop autoimmune thyroid disease because a gene on X chromosome may be responsible for the occurrence of autoimmune thyroid disease. Moreover, both 21-OHD and GD can lead to high level of testosterone, thus we should keep in mind to test chromosome and thyroid function in 21-OHD patients to avoid misdiagnose or missed diagnosis. To the best of our knowledge, this is the first report of SV 21-OHD patient combined with 47, XXX and Graves disease.

## 1. Introduction

Congenital adrenal hyperplasia (CAH) is a group of autosomal recessive disorders featured with deficiency in enzymes responsible for synthesizing steriods of the adrenal cortex.^[1,2]^ The most common form of CAH is 21-hydroxylase deficiency (21-OHD). The notable feature of 21-OHD is the increasing of serum 17 hydroxyprogesterone (17-OHP) and accumulation of adrenal androgen. 21-OHD can be classified into 2 forms according to the residue 21-OH activity: classic CAH has complete enzyme inactivation and severe hyperandrogenism symptoms including classic simple virilizing (SV) form and salt-wasting (SW) form while non-classic CAH (NC CAH) has a variety of clinical manifestations due to its variable levels of 21-OH activity.^[3]^ SV form is characterized by genital ambiguity in female patients with adequate aldosterone secretion while SW form is characterized by impaired mineralocorticoid synthesis.

47, XXX, also called triple X syndrome or Trisomy X, is a genetic disorder characterized by an extra X chromosome in female and is the most common form of sex chromosome variation. However, this disease is often misdiagnosed because most patients have no or very mild symptoms and chromosome detection is not performed in everyone.

Grave’s disease (GD) is an autoimmune disorder that causes hyperthyroidism and hypermetabolic symptoms. In some cases, muscle weakness with amenorrhea can be the initial signs of GD in female and can be easily ignored or wrongly diagnosed.

We report a female diagnosed with SV 21-OHD combining with GD, chromosome 47, XXX, which was not reported so far. As 21-OHD is a kind of disease involved in the lack of steriods, GD may increase the consumption of steriods, which leads to extra testosterone, and at last amenorrhea. It should be noted that 21-OHD, 47, XXX and GD can all have symptoms of amenorrhea to some extent, whereas the first 2 also exhibit external genitals dysplasia. Furthermore, 21-OHD and GD are both associated with elevations of testosterone and virilization to different degrees. Therefore, doctors are advised to pay close attention to screening for related diseases in the process of diagnosis since once a diagnose is made, presence of other 2 illnesses which share similar symptoms with it can be easily ignored.

## 2. Case report

**Figure 1.**
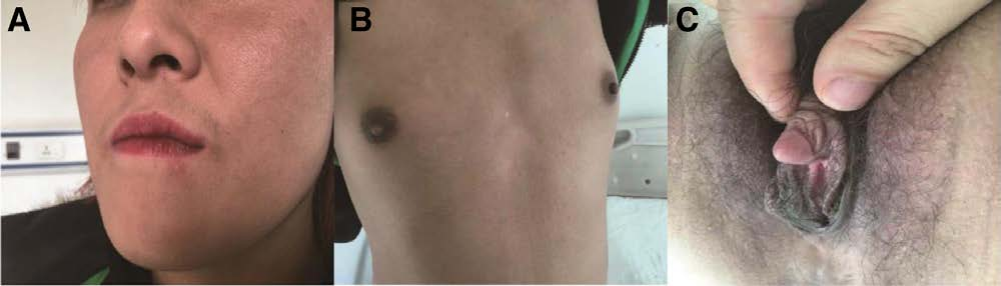
(a) excess hair above the lips; (b) undeveloped breasts with areola hyperpigmented; (c) eclitoral enlargement and male pubic hair distribution, labium hyperpigmented.

A 24-years-old female of Han ethnicity was admitted to the endocrinology department complaining of absence of menses for half a year. The patient didn’t noticed her enlarged clitoris until she was 17 years old and denied any medical, family, and psychosocial history (including co-morbidities and relevant genetic information). Her menarche was 16 years old and the final height was 163 centimeter. She was diagnosed with GD 2 months before admission to our hospital due to palpitation, heat intolerance, muscle weakness. The parameters of the patient were as follows: weight 60 kg, height 163 cm, body mass index 22.5 kg/m^2^, Blood pressure 114/82 mm Hg, pulse 96/ minute. Physical examination revealed the patient with moist skin, finger tremors and mildly diffused goiter. She also had excess hair above the lips (Fig.1 a), hairy abdomen and legs, deep voice and undeveloped breasts with areola hyperpigmentation (Fig.1 b). Genital examination revealed clitoral enlargement and male pubic hair distribution (Fig.1 c). There was no labial fusion or common urogenital sinus.

### 2.1. Laboratory assessments

Laboratory tests found free Triiodothyronine 10.01 pmol/L (3.1–6.8 pmo/L), Free thyroxine 34.43 pmol/L (10–23 pmo/L), Thyroid stimulating hormone 0.005 μIU/mL (0.27–4.2 μIU/mL), testosterone 15.1 nmol/L (0.1–1.67), 17-OHP 11360 ng/dL (300–2340 ng/mL), urine 17-ketosteriod 63.7 mg/24 hours (6.0–25.0mg/24 hours), sodium 135 mmol/L (137–147 mmol/L) and potassium 3.99 mmol/L (3.5–5.5 mmol/L) (Table 3). In order to rule out CAH, medium dose dexamethesone suppression test was conducted. The baseline serum 17-OHP and 24 hours urine 17-ketosteriod was preserved at the first day and later used as reference. The patient was then given dexamethesone 0.75 mg every 6 hours for the next 5 days. Urine and blood samples were taken on the 3rd and 5th day during the test. The inhibition rate of the 3rd day and the 5th day was more than 50% (Table 1). Furthermore, we conducted corticotropin stimulation test – the gold standard of CAH diagnosis. The results are: serum adrenocorticotropic hormone level was significantly increased compared to baseline, but the cortisol didn’t change much. The outcome of corticotropin stimulation test are shown in Table 2.

**Table 1:** Medium dose dexamethesone suppression test.

Parameters	Base	The third day	The fifth day	Reference range
17-OHP (ng/mL)	113.6	0.48	0.53	0.3–2.34 ng/mL
17-KS (mg/24h)	63.7	22.2	25.2	6.0–25.0 mg/24 h

17-OHP: 17 hydroxyprogesterone, 17-KS = 17-ketosteriod.

**Table 2:** Corticotropin stimulation test.

parameters	base	After stimulation	Normal range
ACTH (pmol/L)	14.9	440.4	1.6–13.9
COR at 8am (nmol/L)	91.17	225.49	171–536
17OHP (ng/dL)	7914	28349	0.3–2.34
DHEA (μg/dL)	319.3	344.1	98.8–340

ACTH = adrenocorticotropic hormone, cor = cortisol, DHEA = dehydroepiandrosterone.

**Table 3:** Endocrine profile after treatment.

Parameters	Before treatment	After treatment 3 months	Reference range
FT3 (pmol/L)	10.01	4.64	3.1–6.8
FT4 (pmol/L)	34.43	12.93	10–23
TSH (μIU/mL)	0.005	5.52	0.27–4.2
ACTH (pmol/L)	20.93	2.73	1.6–13.9
Cor at 8am (nmol/L)	424.3	52.5	171–536
Tes (nmol/L)	15.1	0.1	0.1–1.67
17OHP (ng/mL)	113.6	1.01	0.3–2.34
17KS (mg/24 h)	63.7	8.8	6.0–25.0

ACTH = adrenocorticotropic hormone, FT3 = free Triiodothyronine, FT4 = Free thyroxine, Tes = Testosterone, TSH = Thyroid stimulating hormone.

### 2.2. Imageological examination

The adrenal gland Computed Tomography (CT) and pituitary magnetic resonance imaging (MRI) revealed no abnormalities. Pelvic ultrasonography showed normal uterine and ovarian structures.

### 2.3. Genetics evaluation

**Figure 2.**
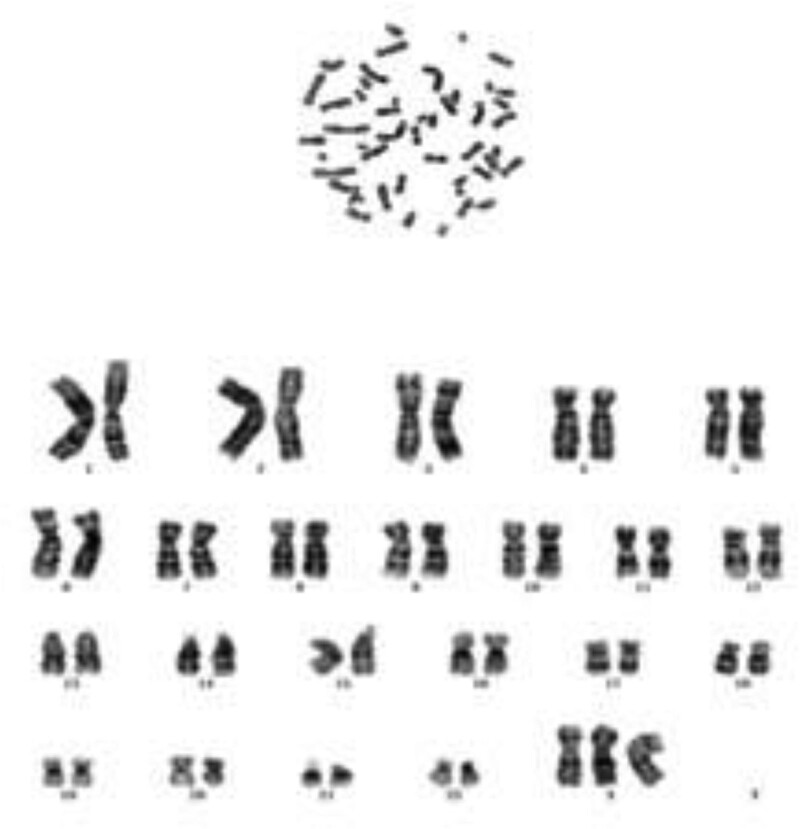
karyotype was found to be 47, XXX, we can see clearly there is an extra chromosome.

**Figure 3.**
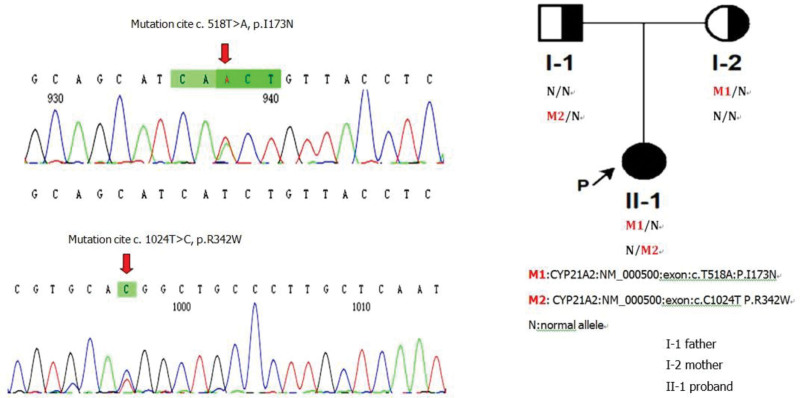
The parents of the patient were carriers for the mutations. The patient had homozygous mutation for the NM_000500:exon4:c. 518 T > A and NM_000500:exon8:c.C1024T).

The patient’s karyotype was found to be 47, XXX (Fig.2). Multiplex ligation-dependent probe amplification and polymerase chain reaction-sequencing was conducted to detect possible gene mutation. There were 2 compound heterozygous mutation in the CYP21A2 gene of the patient. The first one was located in position 519, A replaced T (NM_000500:exon4:c.518 T > A). The second one was in position 1025, T replaced C (NM_000500:exon8:c.C1024T). We can learn from the results that the parents are both carriers. The patient had a healthy elder brother however he refused to do genetic screening (Fig.3).

### 2.4. Follow-up

We confirmed the coexistence of SV 21-OHD, GD and 47, XXX. At first, the patient was given 10 mg methimazole twice a day as well as 5 mg predisone in the morning and 2.5 mg in the evening. After a year of regular medication and reexamination, she got a regular menstruation and thyroid function and now is taking 2.5 mg prednisone twice a day. The results are expected: testosterone declined to 0.1 nmol/L (0.1–1.67 nmol/L) and 17-OHP get back to normal level: 1.01 ng/mL (0.30–2.34 ng/mL), (Table 3). However, her enlarged clitoris has not narrowed.

## 3. Discussion

The patient visited us for abnormal thyroid function and amenorrhea. First, we gave the patient methimazole and then her thyroid function returned to normal. Second, considering her extremely high testosterone level, enlarged clitoris and hairy skin, we tested 17-OHP and found it abnormally elevated. Hence, we did corticotropin simulation test which was the gold standard for 21-OHD diagnosis, the result showed that 17-OHP rose to 28349 ng/dL from 7914 ng/dL and was consistent with the diagnosis of classical CAH. In the meantime, medium dose dexamethasone suppression test was also conducted, the results revealed 17-OHP was suppressed more than 50% in 3rd and 5th days, meaning the excess testosterone came from adrenal gland instead of ovarian tumor. Besides, gene tests also confirmed there were 2 compound heterozygous mutations in the CYP21A2 gene of our patient.

Actually, CYP21 mutations can be divided into 3 types according to the level of 21-OH activity.^[4–7]^ The first category, including deletions or nonsense mutations, inactivates the activity of 21-OH entirely and is most relevant to SW form. The second category, mainly consisting of the missense mutation Ile172Asn (I172N) or I173N, only has 1% to 2% of normal activity and is usually found in SV patients with normal aldosterone. The last category is associated with the NC CAH, it contains mutations which has 20% to 60% of normal activity of 21-OH, such as Val281Leu (V281L) and Pro30Leu (P30L).^[8,9]^ Our patient has I173N site mutation and is consistent with these studies. However, R341 site mutation which is usually found in NC CAH patients appeared in our SV 21-OHD patient. Hence more functional verification in more patients should be done to prove R341’s role in different form of 21-OHD since it is so rare that few articles report it.

In thyrotoxic state, a healthy person could maintain a normal serum cortisol level, while some patients may exhibit fatigue, anorexia due to relatively insufficient cortisol caused by excess thyroid hormone, even placing them at risk for adrenal crisis.^[10]^ For example, Nobuyuki Takasu^[11]^once reported a case that Graves’ hyperthyroidism caused an adrenal crisis in a patient with previously unrecognized non-classical 21-hydroxylase deficiency. In fact, thyroid hormone accelerates the metabolism of cortisol through the effects on the activity of 11βHSD and 5α-reductase enzymes.^[12]^ The sodium level of the patient in our case is slightly lower (135 nmol/L), because too much thyroid hormone in the circulation increase the consumption of cortisol and then affect the electrolyte balance. Likewise, if there is any fatigue, poor appetite or other manifestations of salt loss, we should evaluate the adrenal function and give the right treatment in time.

Moreover, the patient has an extra X chromosome which is called 47, XXX or triple X syndrome and it was first reported in 1959.^[13]^ Though most 47, XXX patients are not associated with a characteristic physical phenotype,^[14–16]^ some of them did show amenorrhea which is similar to CAH. In addition, when a diagnosis of 47, XXX is made, 17-OHP and testosterone is advised to be tested in these patients with virilized external genetalia. In the light of this close relationship, we need to figure out whether 47, XXX and CAH had correlations and the possible mechanisms between them. However there have been few reports on the coexistence of 47, XXX and CAH, except Slim K in 2004^[17]^ reported a triple X syndrome patient with 11β-hydroxylase deficiency and it was the first case of CAH with triple X syndrome. On the contrary, extensive literature on the coexistence of CAH and Turner syndrome have been reported,^[18–27]^ especially Lirriza D^[28]^ have found in Turner Syndrome (TS) patients, the frequency of 21-OHD was extremely higher (21.6%) than that of the general Italian population, which implies patients with Turner syndrome are somehow likely to combine with 21-OHD. As a result, this kind of coexistence has made it difficult for doctors to make a proper diagnosis for they often share similar characteristics, so all the CAH patient are recommended to undergo chromosome inspection regularly. To the best of our knowledge, it was the first case that 47, XXX and 21-OHD appearing in our patient at the same time, but the possible mechanisms that whether 21-OHD are prone to combine with 47, XXX has not been reported yet. In addition, chromosome abnormality such as turner syndrome and triple X had higher frequencies to combine with autoimmune thyroid disease according to Elsheikh and Rhodes et al.^[29–32]^ They believed that X chromosome plays an important role in the maintenance of immuno competence through a gene on the long arm of the X chromosome. In our case, the patient was found to have triple X and autoimmune thyroid disease. Therefore, thyroid function should also be tested as an regular test for patients with abnormal chromosome to avoid hypothyroidism in the future, yet there was no evidence that 21-OHD are easily to combine with autoimmune disease. Additionally, amenorrhea or oligomenorrhea can both occur in triple X, 21-OHD and Graves disease, of which 21-OHD and Graves disease are more likely to have high testosterone level. In specific, researches have concluded that thyroid hormones regulate bioavailability of sex hormones and affect some sexual organs. As free thyroxine increases and thyroid stimulating hormone correspondingly decreases, sex hormone binding globulin (SHBG) concentration increases, and the total testosterone and estradiol concentrations increase. Therefore, it is very easy for doctors to make improper or missed diagnosis when they only considering one possible cause.

In conclusion, karyotyping test should be recommended as an necessary item in 21-OHD patients, for 21-OHD are easily to combine with chromosome abnormality and both of them can lead to amenorrhea or oligomenorrhea. Furthermore, clinicians should not forget to test thyroid function in patients with irregular chromosome, because these people are more likely to suffer from autoimmue thyroid disease. At the same time, we should bear in mind that GD and 21-OHD are both associated with elevated testosterone and corresponding symptoms as well as pay attention to electrolyte in patients with 21-OHD, especially those patients with hyperthyroidism for they are likely to lead to electrolyte disturbance and adrenal crisis. However, this is the first report of SV 21-OHD patient combined with 47, XXX and Graves disease and we haven’t found any genetic association between them.

## Author contributions

**Conceptualization:** Dong Liang, Minmin Han, Linxin Xu.

**Data Curation:** Yi Zhang, Jianhong Yin.

**Funding acquisition:** Yunfeng Liu, Yi Ren

**Investigation:** Minmin Han, Linxin Xu.

**Software:** Dong Liang, Yi Ren.

**Validation:** Yunfeng Liu.

**Writing – original draft:** Dong Liang.

**Writing – review & editing:** Yunfeng Liu, Jing Yang, Linxin Xu, Minmin Han.
